# Fit Is it for Longevity and Prevention of Multimorbidity

**DOI:** 10.1016/j.jacadv.2025.102199

**Published:** 2025-10-08

**Authors:** Carl J. Lavie, Charles Faselis, Peter Kokkinos

**Affiliations:** a Department of Cardiovascular Diseases, John Ochsner Heart and Vascular Institute, Ochsner Clinical School-The University of Queensland School of Medicine, New Orleans, Louisiana, USA; b Veterans Affairs Medical Center, Washington, District of Columbia, USA; c George Washington University School of Medicine, Washington, District of Columbia, USA; d Department of Kinesiology and Health, School of Arts and Sciences, Rutgers University, New Brunswick, New Jersey, USA

**Keywords:** cancer, cardiorespiratory fitness, cardiovascular disease, exercise, multimorbidity, physical activity

**Figure:**
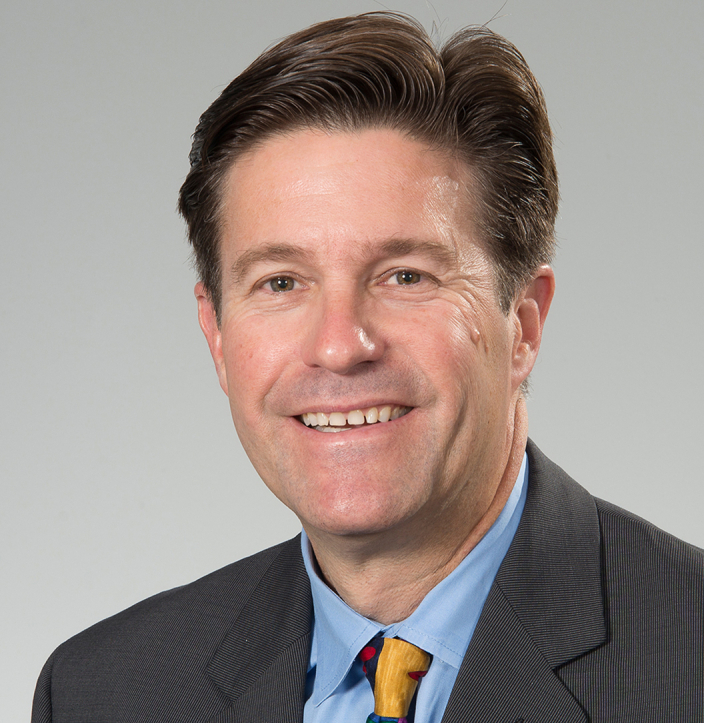

During the last few decades, we and others have demonstrated an inverse and graded association between cardiorespiratory fitness (CRF) assessed objectively by a standardized exercise test, and the incidence of chronic disease, certain cancers, type 2 diabetes mellitus, dementia, and all-cause mortality.^1^, ^2^, ^3^, ^4^, ^5^, ^6^, ^7^, ^8^ In fact, CRF may be perhaps one of the most important risk factors (or “risk marker”) for cardiovascular (CV) disease (CVD) and overall prognosis, as patients with a major CVD risk factor but with relatively high levels of CRF generally have a better prognosis than patients without that risk factor but with low CRF.^2^ More recently, studies have supported the protective role of CRF for even non-CVD and noncancer survival,^9^^,^^10^ and CRF is highly protective for CVD and all-cause mortality in patients at high risk for COVID-19^11^ as well as for those with chronic kidney disease.^12^ Most recently, we have demonstrated that high levels of CRF are also associated with lower risk of developing colon cancer.^13^

The modern triumph of extended human lifespan brings with it an emerging clinical reality: the increasing prevalence of multimorbidity—the coexistence of 2 or more chronic conditions within an individual. With more than 80% of those over 85 now affected by multimorbidity, its rising burden presents both a public health challenge and a pressing clinical concern.^14^ Strategies that delay or reduce the accumulation of chronic conditions are essential not only to improve individual outcomes but also to reduce strain on the health care system.

In this context, the study by Xu et al^15^ in this issue of *JACC: Advances* represents a timely and important contribution to our understanding of CRF as a potential preventive determinant of multimorbidity. Using data from over 38,000 initially disease-free participants in the UK Biobank followed for up to 15 years, the authors demonstrate that higher CRF is associated with a significantly lower risk of developing multimorbidity, a delay in its onset, and a slower trajectory in the accumulation of chronic diseases. These findings extend prior literature linking CRF to individual disease outcomes and offer a broader, integrated view of how physiological fitness may modulate age-related disease clustering.

## Context and contribution

The current study by Xu et al^15^ builds on this foundation by showing that higher CRF at midlife may serve as a buffer against the cumulative burden of chronic disease, what might be called the physiological trajectory toward multimorbidity.

Notably, the authors report a dose-response association between CRF and multimorbidity risk: those with high CRF had a 21% lower risk of developing multimorbidity compared to those with low CRF and experienced a delay of over 1 year in its onset. Furthermore, individuals with higher CRF exhibited slower accumulation of metabolic, CV, and neuropsychiatric diseases.^15^ These observations suggest that CRF may act as a system-wide modulator of chronic disease progression.

## Strengths and limitations

Several methodological strengths bolster the credibility of these findings. First, the use of a large, well-characterized cohort with long follow-up and linkage to medical records minimizes recall bias and allows for temporality in inference. Second, the authors performed age- and sex-standardization of CRF and adjusted for a wide range of covariates, including socioeconomic status, smoking, alcohol use, and physical activity (PA), increasing confidence in the independent role of CRF. Third, sensitivity analyses, excluding early incident cases and accounting for the competing risk of death, demonstrated robustness of the results.

Despite these strengths, several limitations warrant mention. As the authors note, the UK Biobank population is known to be healthier, less racially and ethnically diverse, and more socioeconomically advantaged than the general UK population,^16^ which may limit generalizability. This concern is amplified when considering applicability to more heterogeneous populations such as those represented in U.S. biobanks, like the Million Veteran Program, which include greater racial, socioeconomic, and geographic diversity and may better reflect clinical realities in the U.S. health care system.^17^ Moreover, individuals with baseline disease or deemed too high risk were excluded from the CRF test, further narrowing the range of clinical vulnerability captured in the sample.

Second, residual confounding from unmeasured factors, such as diet, medication adherence, or genetic susceptibility, cannot be fully excluded. Third, while the authors adjusted for self-reported PA, it remains difficult to disentangle the effects of CRF from habitual PA in observational studies, as the 2 are closely linked: individuals who engage in regular exercise tend to have higher CRF, making it challenging to isolate whether CRF per se or the underlying behavioral pattern is driving the observed associations. Finally, although many assessments of CRF are associated with subsequent prognoses,^18^ they estimated CRF using a 6-minute submaximal exercise test, as opposed to more precise assessments from a maximal stress test or using the “gold standard” cardiopulmonary exercise testing with gas exchange.

## Biological mechanisms and future research

Further research is also needed to better understand the biological mechanisms connecting CRF and lower risk of multimorbidity. Previous and recent studies have linked high CRF to lower levels of chronic inflammation, enhanced immune function, reduced visceral adiposity, and improved antioxidant capacity and emerging data from both animal models and human studies suggest that these benefits are mediated through organ-specific molecular adaptations to exercise, further strengthening the plausibility of CRF as an independent modifier of disease risk.^1^, ^2^, ^3^, ^4^, ^5^, ^6^, ^7^^,^^19^^,^^20^

These mechanistic findings offer a promising foundation for future translational work. Randomized trials are needed to establish whether interventions that increase CRF, particularly in midlife, can causally reduce the burden of multimorbidity. The integration of CRF with emerging biomarkers, including genomics, proteomics, and metabolomics, may also enable more personalized preventive strategies in aging populations.

## Implications for clinical practice and public health

The findings of this study suggest that CRF may be a valuable addition to clinical risk stratification tools, providing a system-wide snapshot of physiological reserve and disease vulnerability. CRF measurement, long relegated to the realm of sports medicine and cardiopulmonary testing, deserves broader consideration in midlife health assessments. Even more importantly, it underscores the value of fitness, beyond weight loss or CVD endpoints, as a holistic preventive strategy against aging-related multimorbidity.

Notably, the study highlights that the association between CRF and multimorbidity was more pronounced in participants under the age of 60, supporting the notion that midlife may represent a critical window for preventive interventions. This aligns with a growing body of evidence suggesting that risk factor modification earlier in life may yield the greatest long-term gains in healthy aging.^21^ Importantly, the health benefits associated with increased CRF are also realized in septuagenarians and octogenarians, across racial groups, and in both genders,^22^ and changes in CRF over time also impact mortality.^23^ Thus, maintaining an active lifestyle should be promoted by health care professionals for all ages.

## Conclusions

In an era of aging populations and escalating chronic disease burden, the findings by Xu et al^14^ offer convincing evidence that maintaining CRF is not merely about adding years to life but adding life to those years. Clinicians and health systems should view CRF as more than a fitness metric, but a barometer of future health, an opportunity for intervention, and a lens through which multimorbidity prevention may be advanced. Clearly, “Fit is It” for longevity and for the prevention of multimorbidity.^24^^,^^25^

## Funding support and author disclosures

The authors have reported that they have no relationships relevant to the contents of this paper to disclose.
